# H-DSAE: a hybrid technique to recognize heart disease

**DOI:** 10.3389/fphys.2025.1563199

**Published:** 2025-06-05

**Authors:** K. Uma Maheswari, A. Valarmathi

**Affiliations:** ^1^ Department of Information Technology, University College of Engineering, BIT Campus, Anna University, Tiruchirappalli, India; ^2^ Department of Computer Applications, University College of Engineering, BIT Campus, Anna University, Tiruchirappalli, India

**Keywords:** DBN, SAE, SVM, heart disease recognition, clinical decision making

## Abstract

Over the years, the number of people who succumbed to heart ailments has increased significantly worldwide. The World Health Organization claims that about 17 million people die each year due to heart disease. High levels of cholesterol and blood pressure are some risk factors. This technology seeks to treat these conditions before they become a problem. Through machine learning, doctors can now make more informed decisions regarding the treatment of patients. Machine learning can assist in reducing the likelihood of a cardiac event. Conventional methods for diagnosing diseases often lead to inaccurate diagnoses and take longer to complete due to human errors. In order to increase the diagnostic accuracy, an ensemble method is used. This method combines various classifiers to achieve highly accurate predictions. Due to the complexity of the task, the researchers decided to use deep learning methods to perform the heart disease classification task. H-DSAE technique utilize Deep Belief Network (DBN), Support Vector Machine (SVM), and Stacked Auto-Encoder (SAE). It was able to extract various heart image representations and achieve an accuracy of 99.2. It also had a sensitivity of 97.5, F-measure of 98.5, and precision of 98.4. The next phase of the project will focus on developing more advanced classification and features algorithms. This will help improve the efficiency of the system.

## 1 Introduction

Quality healthcare is a vital aspect of providing effective services to patients at affordable costs. This can be accomplished by employing the proper tools and procedures. Poor decisions made by doctors can lead to disastrous results. This is because they rely on their gut feeling instead of the data gathered from the database (Sakr and Elgammal, 2016). This practice leads to the accumulation of unnecessary medical costs and biases that affect the quality of healthcare provided to patients. According to a recent study, utilizing clinical decision support may help enhance the quality of healthcare by reducing medical errors (Saposnik et al., 2016). Most hospitals use an information system to manage their patient data. Unfortunately, this system rarely provides the necessary support for clinical decision making (Lintern and Motavalli, 2018). The research seeks to develop a method that can identify cardiac problems in patients with historical data. This would lead to a reduction in medical examinations and a boost to the quality of healthcare.

According to World Health Organization (WHO) cardiovascular diseases are responsible for most deaths worldwide. It states that millions of people are killed annually due to this condition (Balakumar et al., 2016). About 80% of deaths caused by cardiovascular diseases are caused by strokes and coronary artery disease. In low and mid-income nations, heart disease is prevalent (Yusuf et al., 2020). High blood pressure and stress are some risk factors that can lead to heart disease. In addition, a person’s lifestyle and genetic makeup can also affect their chances of developing this condition (Han and Lean, 2016). Big data mining is a process utilized to retrieve information from large databases. It can help to predict the likelihood of heart disease (Repaka et al., 2019a). A comparison of the several classification techniques is performed. In recent years, the utilization of neural network models has advanced dramatically (Arabasadi et al., 2017). Through deep learning techniques, healthcare professionals can now develop effective disease classification systems. (Alafif et al., 2021; Esteva et al., 2019; Yang and Gao, 2018; Rudin, 2019; Martino et al., 2020).

Not having enough collected Electronic Health Record (EHR) data is a major challenge for healthcare analysis. Also, the huge imbalance in the datasets can affect the predictive models for healthcare in heart disease prediction (Norori et al., 2021; Bolón-Canedo and Alonso-Betanzos, 2019). We introduce a resampling technique that can adapt to the output class distribution and a framework that can classify various types of heart disease (Baccouche et al., 2020). Numerous data sets and models have been developed to study the various risk factors associated with heart disease (Rezaee et al., 2020). However, these models and datasets are not yet powerful enough to identify the most important risk factors (Song and Ying, 2015). Using feature learning techniques, it is feasible to compile a list of important characteristics that can be easily categorized (Litjens et al., 2017). This method can help a model detect and classify the data efficiently. Auto encoders are also known to extracting hidden representations from data that have gained increased attention due to their performance (Ma et al., 2020).

The study sought to develop a way to predict the course of a cardiovascular condition. Through statistical analysis, the model was able to identify the various factors that can affect a person’s health. The model was then developed using the Deep Belief Network framework. The statistical techniques used in the study were utilized to analyze the various variables. As the network’s depth increases, the number of data transformations increases, which allows the model to perform more complex analysis. Unlike traditional statistical methods, deep learning can handle immense amounts of information. However, one of its main limitations is that it can’t guarantee the existence of global minimum value. The suggested model takes advantage of both SVM and deep learning. By combining the two, it can effectively detect heart disease. The proposed model can also perform good generalization when the parameters are properly configured. It can also model any training set provided that the appropriate kernel is used. The paper is divided into five sections. The second one reviews the related works, while the third one defines the framework and concepts utilized in the experiment. The fourth section presents the study’s findings, and results of the simulation. The fifth section summarizes the work.

## 2 Related works

Katarya and Meena (2021) A study found that being overweight significantly increased the likelihood of heart disease. The researchers were able to use machine learning to analyze different aspects of this health issue. They then used a random forest algorithm to improve their analysis’s accuracy.

Ali et al. (2021) The researchers evaluated a massive database of information about heart disease to find the most efficient methods for predicting the condition. They then ranked these features using a scale of importance to find out which algorithms were most useful.

Kavitha et al. (2021) The researchers used the Cleveland database to develop an algorithm for machine learning. They tested the model’s accuracy by performing an experiment and found it to be capable of accurately predicting the heart conditions with an accuracy of almost 88 percent. This demonstrates how deep learning can be utilized in predicting various medical conditions.

Jindal et al. (2021) The study used a prediction system to identify the individuals who are more prone to developing heart conditions. The researchers utilized logistic regression and K-Nearest Neighbors (KNN) to analyze the data. They were able to classify the individuals and determine the presence of various illnesses. The researchers were also able to predict with high accuracy which diseases would be discovered.

Mehmood et al. (2021) The researchers presented the CardioHelp, a machine learning tool that can predict an individual’s likelihood of experiencing a heart attack. By being able to identify such conditions at an advanced stage, it could prevent them from causing harm. The evaluation of the suggested method revealed that it was more effective than current models.

## 3 Proposed methodology that utilized the DBN-SAE-SVM to predict heart disease

**FIGURE 1:**
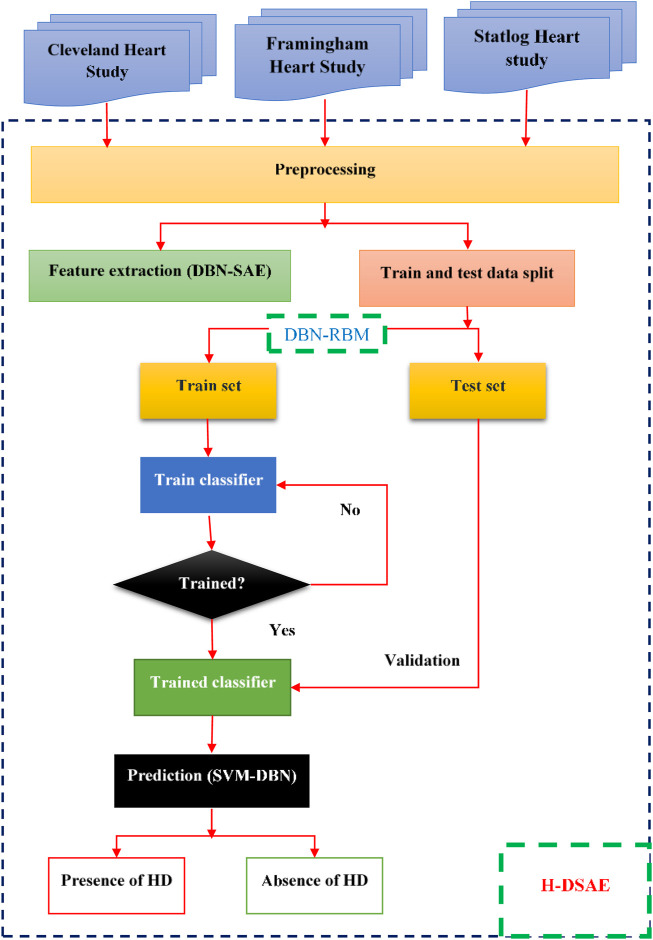
Proposed H-DSAE architecture.

One of the most common conditions that people deal with globally is heart disease. Being aware of this disease’s symptoms and early detection are key to treating it. The objective of this system is to analyze and interpret the data collected from patients’ health records in a timely and accurate manner. It can also perform various clinical decisions based on its deep learning network. The H-DSAE framework can be easily integrated into healthcare facilities to diagnose heart conditions. The training’s outcome is evaluated using performance metrics. The structure of the framework is shown in Figure 1. It aims to create a system that can identify heart diseases.

### 3.1 Datasets

The study uses three heart study datasets, namely, the Cleveland heart study dataset (2025), the Framingham heart study (2025) and Statlog data set (2025). The Cleveland Heart Study has a total of 304 samples and 14 attributes. The other data set, the Framingham Heart Study, has a total of 4238 samples and 16 features. These include behavioral risk factors and medical risk factors. The Statlog data set contains 270 instances. These instances are composed of two categories, ordered multiclass, and non-ordered multiclass variables.

The validation and training datasets are divided into 30% and 70% respectively. The missing values are computed through mean imputation. Data imputation is a technique that uses the existing details in a dataset to create a substitute value. It's commonly used to preserve the most important information in a data set.

### 3.2 Deep Belief Network and Auto encoder

**FIGURE 2:**
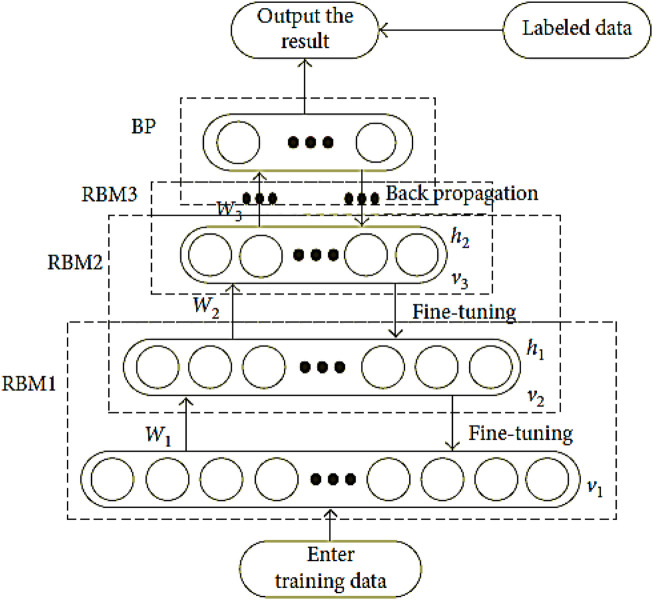
The training flow chart for DBN.

The DBN is a type of deep learning model that can be used to stack various RBM. The DBN is a combination of latent and stochastic variables. It can be regarded as a special type of Bayesian generative model. A neural network is composed of various layers that are connected to the inputs. The outputs of these layers then go to the inputs of the next one in a backward manner as shown in Figure 2.

The Auto-Encoders method can be utilized to reduce the input’s dimensionality. The goal of a structured analysis is to capture a hierarchy of groups and a partial-total decomposition of an input. In order to achieve this, the first and second layers of a program tend to learn first-order functions in the raw input. Support vector machines are also known to provide strong generalization capabilities. They can learn from small samples and can solve the problem of feature division.

This process seeks to develop a way to predict an individual’s risk of heart disease. The RBM model shows that the units in the layers have no connection to each other. The input vector is the configuration’s visible vector. The characteristic that emerges from the visible vector is the hidden vector.

$$E\left(v,h\right)=-\sum_{i=1}^{I}v_{i}a_{i}-\sum_{j=1}^{J}b_{j}h_{j}-\sum_{i=1}^{I}\sum_{J=1}^{J}h_{i}v_{i}w_{ij}$$ (1)

In Equation 1, the connection weight of the two layers is ij and ai. The visible layer (ij) unit is i, while the hidden layer (ai) unit is j. The difference between these two is that the former is visible, while the latter is hidden.

The following Equation 2 illustrates the visible layer’s conditional distribution.

$$p\left(h_{j}\left.=1\right|v\right)=\sigma (\sum_{i}w_{ij}v_{i}+b_{j}$$ (2)

Following are the conditions for the hidden layer distribution (Equation 3)

$$p\left(v_{j}\left.=1\right|h\right)=\sigma (\sum_{j}w_{ij}v_{j}+b_{i}$$ (3)

The sigmoid function is often utilized as the activation procedure in Equation 3.

The distribution of the hidden and visible elements is shown by their joint probability in Equation 4:

$$p\left(v,h\right)=\frac{1}{z}e^{-E\left(v,h\right)}$$ (4)

Where Z is calculated by adding all feasible visible and hidden vectors as in Equation 5:

$$z=\sum_{v}\sum_{h}e^{-E\left(v,h\right)}$$ (5)

For the calculation of the hidden layer, the input v must contain the number of digits that contain the hidden layer. The hidden layer can be computed using Equation 2. It can then be reconstructed using Equation 3. The training process for deep learning involves mining deep representations of features from multiple RBMs. The DBN is formed by stacking multiple layer as presented in Equations 6–8.

$$\nabla w_{ij}=\varepsilon \left({\langle v_{i}h_{j}\rangle }_{p\left(h|v\right)}-{\langle v_{i}h_{j}\rangle }_{recon}\right)$$ (6)

$$\nabla b_{j}=\varepsilon \left({\langle h_{j}\rangle }_{p\left(h|v\right)}-{\langle h_{j}\rangle }_{recon}\right)$$ (7)

$$\nabla a_{j}=\varepsilon \left({\langle v_{i}\rangle }_{p\left(h|v\right)}-{\langle v_{i}\rangle }_{recon}\right)$$ (8)

The symbol “<•>” in the reconstruction function shows the expectation of an implied partial derivative in the model distribution.

The DBN network’s fine-tuning phase aims to improve the network’s feature extraction effect. This procedure is carried out to minimize the number of diagnostic mistakes that can occur when using a network.

The process of extracting and reducing dimension features in SAE involves two steps. The first one is the encoding and decoding of the data as in Equation 9.

$$h=f\left(x\right)=g\left(WX+b_{1}\right)$$ (9)

The hidden units’ descriptions are included in the data that was reconstructed during the encoding process as in Equation 10.

$$z=g\left(vh+b_{2}\right)$$ (10)

In Equation 10, the data vector Z is the high-dimensional representation of the data, while the output from the hidden layer is the low-dimensional one. The optimal weights for the W and b1 bias vectors can be achieved through back propagation. In this technique, the output values are derived from the input values using the identity function’s y(i)-x(i). The activation function in Equation 11 is then used to generate the output values from the hidden and output layers.

$$T=\frac{1}{2m}{\sum_{i=1}^{m}\left\|x_{i}-{\hat{x}}_{i}\right\|}^{2}+\frac{\lambda }{2}(\sum_{k,n}W^{2}+\sum_{n,k}V^{2}+\sum_{K}b_{1}^{2}+\sum_{n}wb_{2}^{2}+\beta \sum_{j=1}^{k}KL\left(\rho \|{\hat{P}}_{j}\right)$$ (11)

The use of SAE techniques helps in minimizing the cost of the data. The first two terms are sum of square errors and decay, respectively. Finally, the weight decay is utilized to improve the prediction of the data’s performance, and lastly, the hidden layer is constrained through the sparsity penalty.

The SVM is a tool that can separate the decision categories from the training mode in high-dimensional space, represented in Equation 12.

$$y_{i}\left(w.x_{i}+b\right)\geq 1$$ (12)

where the normal vector of a hyper plane is w, while its offset is b.

It can do so by taking the data collected during a training session and estimating the optimal training solution for that particular hyper plane as presented in Equation 13.

$$\min_{w,b}\frac{1}{2}{\left\|w\right\|}^{2}$$ (13)

The procedure for making a classification decision is expressed in terms of Equation 14:

$$f\left(x\right)=sign\left(w^{*}.x+b^{*}\right)$$ (14)

A kernel function is a commonly used tool to construct the classifier for non–linearly separable samples, such as those with high-dimensional space. It can then map the samples to the space, and solve linearly related problems as in Equation 15.

$$\min_{w,b\xi 2}\frac{1}{2}{\left\|w\right\|}^{2}+C\sum_{i=1}^{N}\xi_{i}$$ (15)

I = 1, 2, N, where N is the number of samples.

The categorization decision function is written as follows Equation 16 in this case:

$$f\left(x\right)=sign\left(C\sum_{i=1}^{N}\xi_{i}+\frac{1}{2}\left\|w\right\|\right)$$ (16)

Linear inseparability is achievable by mapping the input’s position to a high-dimensional area and by introducing the K kernel function. After the linear classification procedure is realized, the SVM algorithm is then utilized to perform the optimal decision as in Equation 17.

$$f\left(x\right)=sign\left(\sum_{i=1}^{N}{a_{i}}^{*}y_{i}K\left(x.x_{i}\right)+b^{*}\right)$$ (17)

The kernel function is shown as K(x, y), while the radial basis function is utilized.

## 4 Result and discussion

This study aims to predict a person’s likelihood of experiencing a heart attack by analyzing statistical data, Python offers many libraries specifically designed for statistical analysis. The data collected were then analyzed and used to generate predictions. The results of the study were then analyzed and compared to the proposed methods. Performance evaluation methods were then used to evaluate the results.

### 4.1 Dataset and features

**FIGURE 3:**
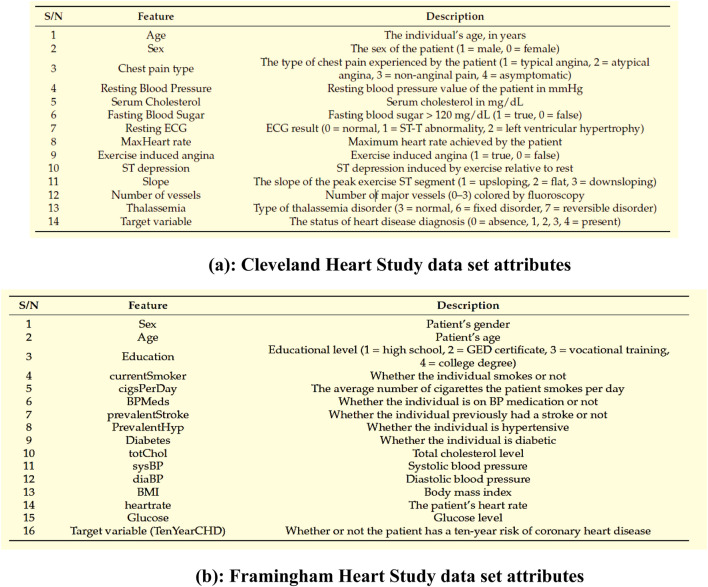
**(a)** Cleveland Heart Study data set attributes **(b)** Framingham Heart Study data set attributes.

Figure 3 shows the Data set attributes of Cleveland Heart Study and Framingham Heart study. Figure 5 shows the heat map of dataset. A heat map is composed of two-dimensional representations of data, which show the values in various colors. It can be useful when plotting the data to identify the most concentrated areas. For instance, Figure 6 shows the age distribution within the dataset, which helps to classify the likelihood of a disease among individuals.

**FIGURE 4:**
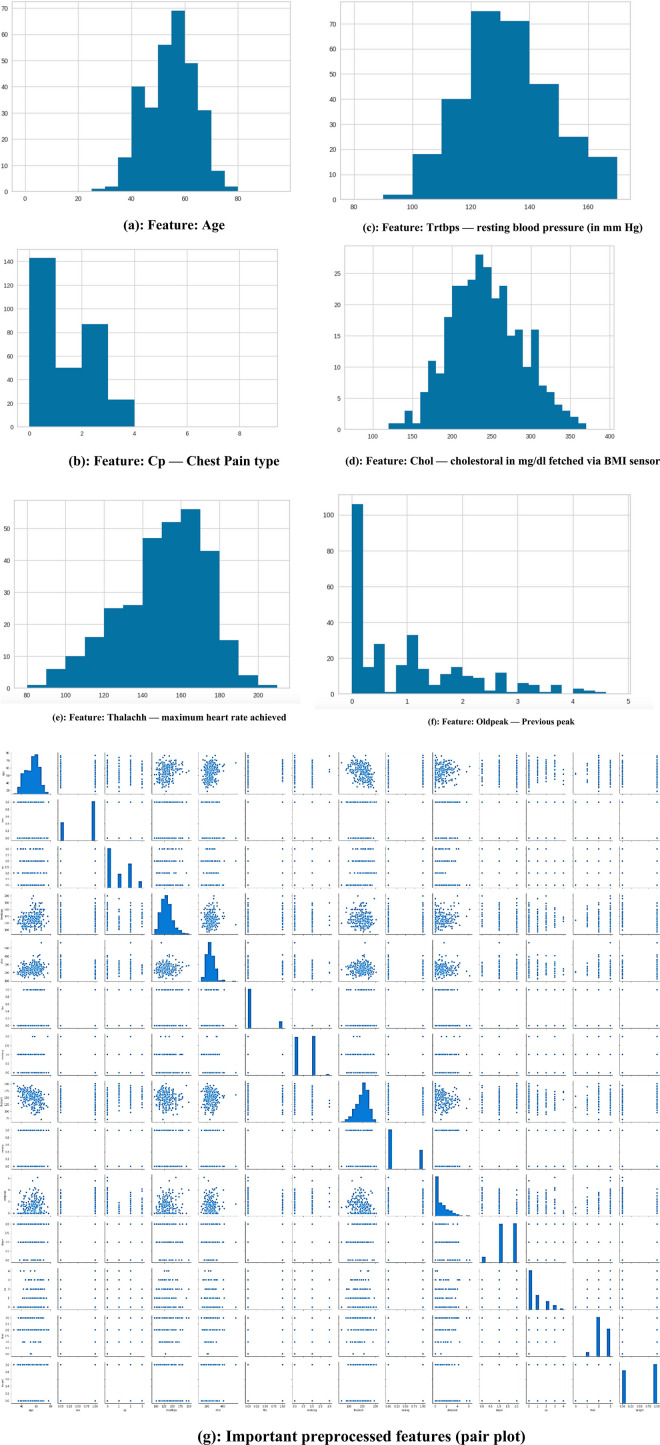
**(a)** Feature: Age **(b)** Feature: Cp—Chest Pain type **(c)** Feature: Trtbps—resting blood pressure (in mmHg) **(d)** Feature: Chol—cholestoral in mg/dl fetched via BMI sensor **(e)** Feature: Thalachh—maximum heart rate achieved **(f)** Feature: Oldpeak—Previous peak **(g)** Important preprocessed features (pair plot).

Figure 3a encapsulates the Cleveland Heart Study data set attributes and Figure 3b encapsulates Framingham Heart Study data set attributes. Preprocessing techniques are then utilized to obtain a balanced dataset. These include scaling, transformation, and handling of various attributes, such as cholesterol level and age. Pre-processing can enhance the quality of a dataset while not directly fixing over-fitting, this makes it easier for a model to generalize. Through the pre-processing of the data, the classifier can improve its performance in predicting outcomes. Figure 4 shows some of the preprocessed features plot as follows. Figure 4a shows age, Figure 4b shows Cp- Chest Pain type, Figure 4c shows Trtbps-resting blood pressure (in mmHg), Figure 4d shows Chol—cholestoral in mg/dl fetched via BMI sensor, Figure 4e shows Thalachh—maximum heart rate achieved, Figure 4f shows Oldpeak—Previous peak. Figure 4g shows the important features that have taken after the preprocess step, the pair plot shows that there is dependency between the variables. The learning model takes into account the training data and then uses the testing data to generate results. Results highlight the distribution of non-cardiac and cardiac patients in the dataset. Age, gender, trestbps, chest pain types, chol, fbs, thalach, bb, ST deperession are some of the features used to identify the heart disease.

**FIGURE 5:**
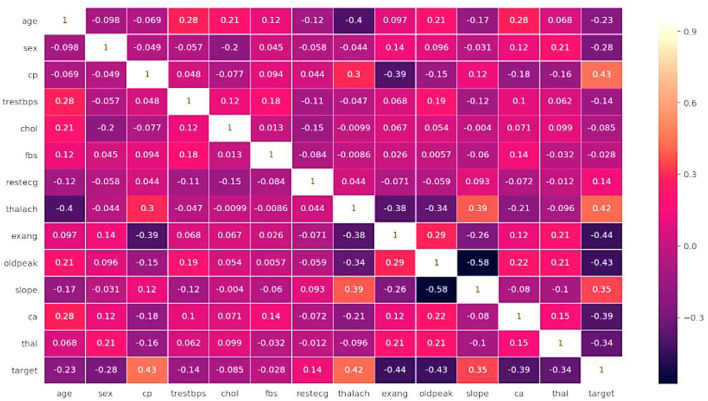
Heat map of dataset.

Figure 5 illustrates the relationship among the attributes of a given dataset using a heat map. A single heat map can quickly summarize the data. More complex ones can be used to analyze the data in more detail. In addition, it can be helpful in identifying the most concentrated data intersections. If we consider sex from X-axis and age from Y-axis, the intersection value be −0.098, which indicated age has a negative impact and if age increase the chance of getting heart disease also increase. If we consider testbps from x-axis and fbs from Y-axis, intersection value is 0.18, which has positive impact if both are kept normal then the possibility of heart disease also decreases.

**FIGURE 6:**
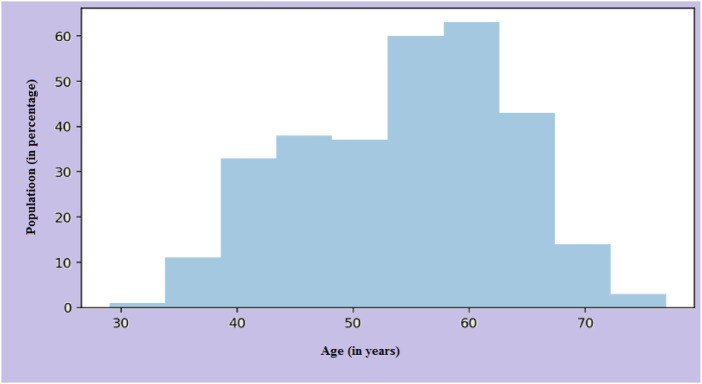
Age distribution on the dataset.

The main risk factor of heart disease is age. With age, the condition becomes more prevalent, which means more mature individuals are affected as presented in Figure 6. The changes in blood vessels observed as people grow older can affect cardiovascular disease risk. A graph or chart can be used to determine the prevalence of cardiovascular disease by age. It can show how this condition changes in different groups. For instance, the distribution might show a rising trend for older adults. Changes in one’s heart or blood vessels are some of the risk factors for heart disease. In addition, age-related issues can also cause this condition.

### 4.2 Prediction results

**FIGURE 7:**
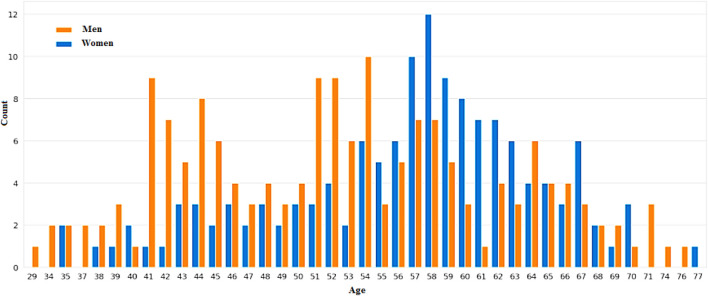
Individuals’ ages and gender determine their elevated risk of heart disease.

**FIGURE 8:**
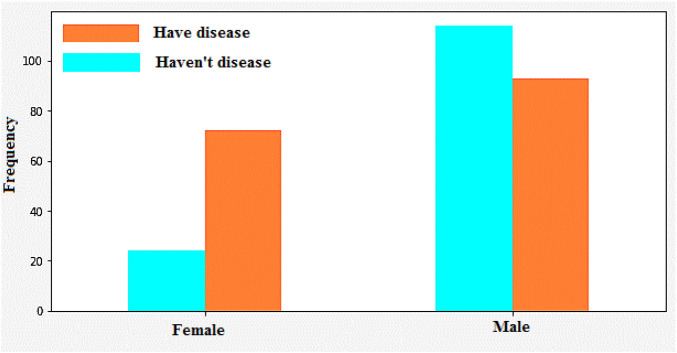
Heart disease identification/classification based on gender.

Figures 7, 8 categorizes the high risk of heart disease based on age and gender. “Orange” indicates men and “Blue” indicates women. The appearance of heart disease is influenced by age, but the manifestation and timing of this condition differ between women and men. For instance, in men, the risk of developing cardiovascular disease increases linearly as one enters old age, while women experience an accelerated increase after menopause (Naftolin et al., 2019). This is due to the protective effects estrogen has on premenopausal women.

#### 4.2.1 Men

As people get older, their heart health becomes more unpredictable. As they get older, men are more prone to experiencing heart failure with a reduced ejection fraction. Men are also more prone to experiencing ventricular arrhythmias. The development of heart disease typically occurs at a younger age for men than women.

#### 4.2.2 Women

After menopause, the risk of heart disease significantly increases. The decline in estrogen following menopause can lead to a higher risk of heart disease. With preserved HFpEF, women are more prone to experiencing heart failure. Women over the age of 75 are also more prone to experiencing hypertension. This condition affects women more than men of the same age.

**FIGURE 9:**
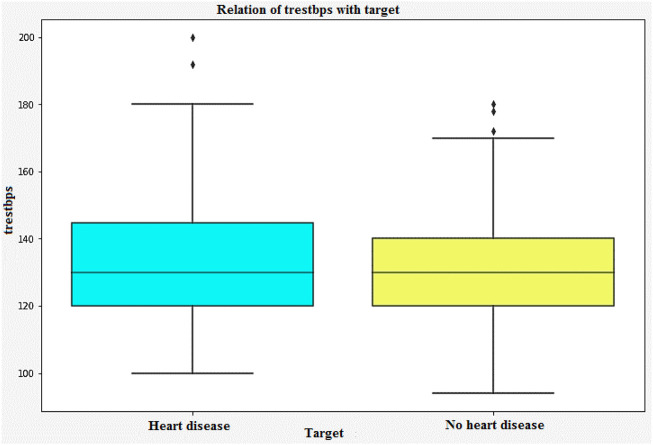
Relation of trestbps with target.

Figure 9 shows that the correlation between the characteristics of a given variable (trestbps) and its target. Resting blood pressure is another component of heart disease prognosis. This test can be used to check if an individual is at high risk of heart disease. High resting blood pressure typically leads to an increased heart disease risk. Other factors such as chest pain and the maximum heart rate (thalach) can also be used to identify potential risk factors.

**FIGURE 10:**
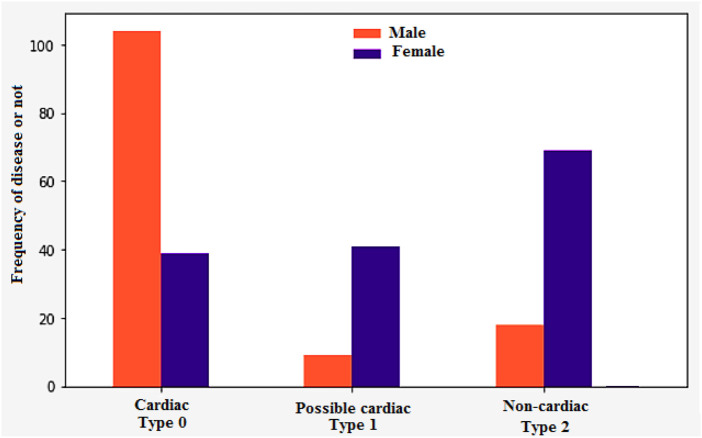
Disease frequency based on chest pain type.

The disease frequency based on the type of chest pain is shown in Figure 10. For instance, “type 0” indicates cardiac, while “type 1” indicates possible cardiac and “type 2” indicates non-cardiac. Patients with type 1 myocardial infarction (MI) experience chest pain ranging from 49% to 91%. On the other hand, those with type 2 MI experience pain ranging from 9% to 62%. This condition, which is often associated with arrhythmia or hypertension, is more common than those with type 1 MI. About 20%–40% of the general population experience chest pain during their lifetime. It is considered a common symptom that most people will experience at some point. About 1.5% of the population visits a primary care doctor for treatment. Various conditions can trigger chest pain. Some of these include: malignant diseases, gastrointestinal disorders, and psychological and psychiatric disorders. Although cardiac disease is the most common cause of chest pain, it is estimated that only a minority of people experience it. Although many people with chest pain have no underlying cause, they still feel anxious about their condition and are prone to experiencing life-threatening symptoms. While it is important to consider the exact cause of chest pain, it is also important to identify other possible causes to get the proper treatment.

**FIGURE 11:**
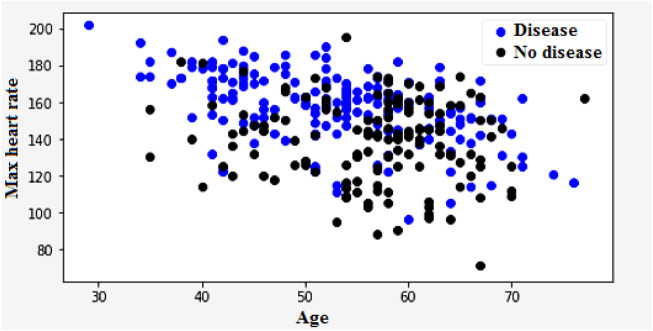
Heart disease classification based on heart rate and age.

In Figure 11, age and heart rate variability are the factors that determine heart disease’s classification. The variability of heart rate is a vital part of the body’s functioning and can be used to identify various abnormalities. Due to the importance of heart rate variability in the diagnosis of heart disease, it has gained increasing popularity in the field of cardiology. The three different ways to classify heart rate are fast, normal, and slow. Resting heart rates that are less than sixty beats per minute are regarded as slow. On the contrary, resting heart rates of over a hundred beats per minute are regarded as fast.

**FIGURE 12:**
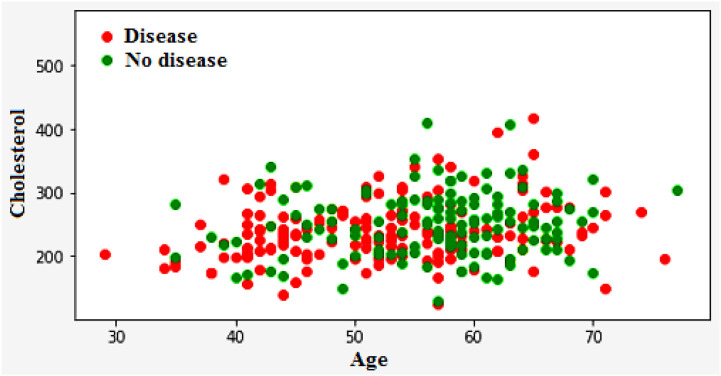
Heart disease classification based on cholesterol and age.

**FIGURE 13:**
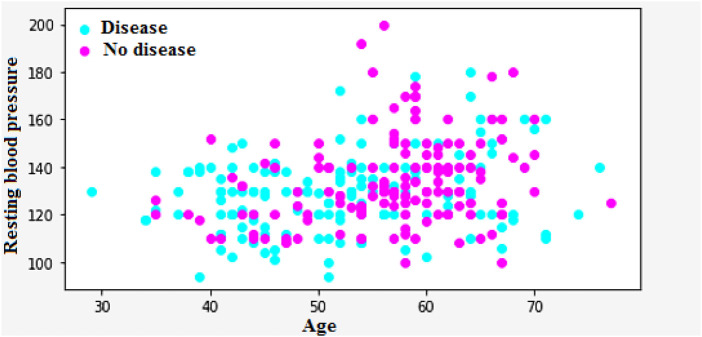
Classification of heart diseases based on the age and Bp.

**FIGURE 14:**
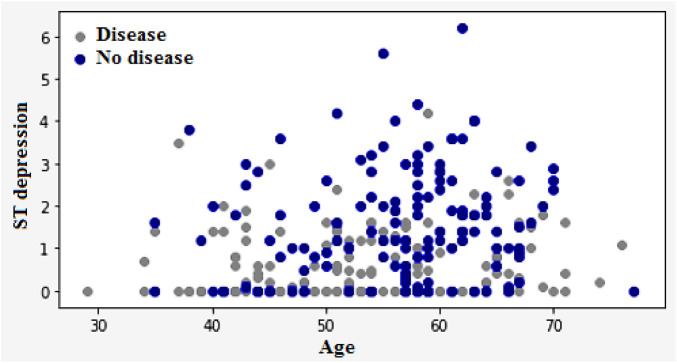
Classification of heart disease based on the age and ST depression.

Figure 12 illustrates the classification of cardiovascular diseases by considering the various risk factors, including cholesterol and age. About one-third of men and over half of women have a higher HD risk due to their age. In women, the higher HD risk is more prevalent in the 50–64 years age group compared with the 25–39 years age group represents in Figure 12. The risk factors that increase the HD risk are as follows: decreased total cholesterol ratio, increased systolic blood pressure, and depression is depicted in Figures 13, 14.

**FIGURE 15:**
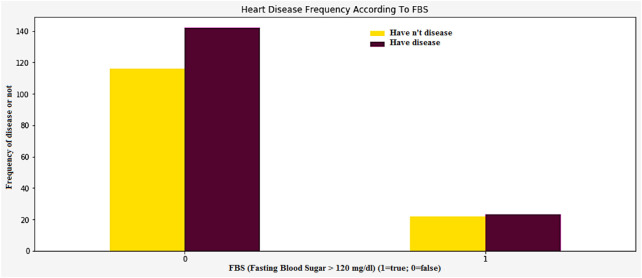
Heart disease frequency according to FBS.

High blood pressure and diabetes are two of the most common causes of heart disease and stroke. Both low and impaired glucose levels are also known to be associated with these conditions. The relationship between low and impaired glucose levels and CVD risk was generally followed by J-shape curves. High glucose levels are known to increase the risk of various cardiovascular illnesses and death. However, the incidence of hemorrhagic stroke did not go up. High glucose levels are known to increase the likelihood of stroke. They were also associated with a higher hazard ratio. The Figure 15 shows the frequency of heart disease according to the FBS.

**FIGURE 16:**
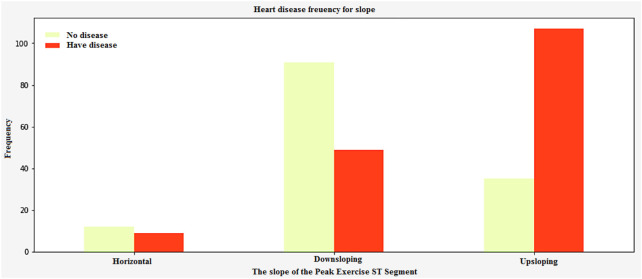
Heart disease frequency curve slope of the Peak exercise ST segment.

In Figure 16, the peak exercise segment’s slope can be affected by factors such as the prevalence of Thalassemia and the number of vessels. The data collected from this study is very important in identifying which patients are most at risk of heart disease. This is an experimental task that can help identify and diagnose various health conditions. The peak exercise segment ST-segment has three different types of slopes. These are upsloping, horizontal, and downsloping. Figure 16 shows the graphical representation of these slopes.

### 4.3 Performance analysis

Performance metrics are tools that measure a system’s speed and accuracy. In this paper, we will use formulas to identify the most common criteria that are used to evaluate a system’s performance.

#### 4.3.1 Precision

The precision of a given data set is expressed as the number of detected data in relation to the relevant data as in in Equation 15.

#### 4.3.2 Recall

Recall analysis are procedures that take into account the values of dependent and independent variables, presented in Equation 16. It can be performed within certain limits.

#### 4.3.3 Accuracy

The most common method used to determine the effectiveness of a classification is by measuring its overall efficiency. Accuracy is measured as presented in Equation 17.

**FIGURE 17:**
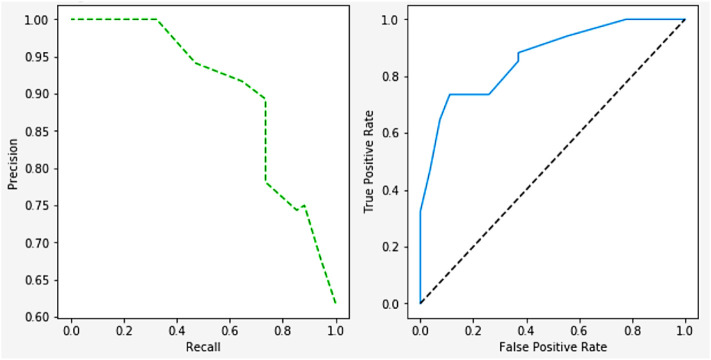
ROC curve on performance.

Figure 17 shows the relationship between the negative and positive rates of predictive models. The ROC Curves also shows the trade-offs between the precision, recall, F-measure, and recall values. The precision is 98.45, recall is 97.55, and the F-measure is 98.51.

**FIGURE 18:**
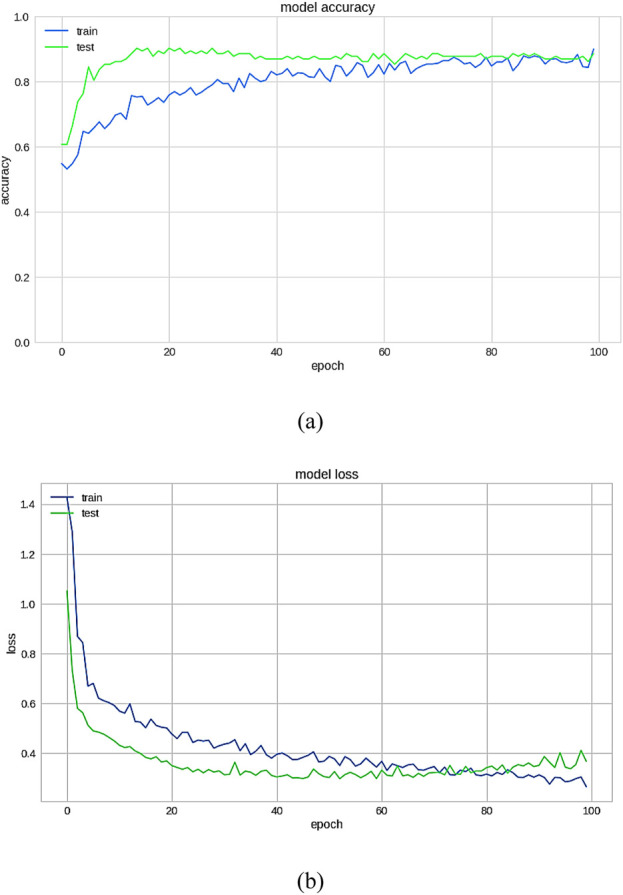
**(a)** Accuracy **(b)** Loss on the test set.

**FIGURE 19:**
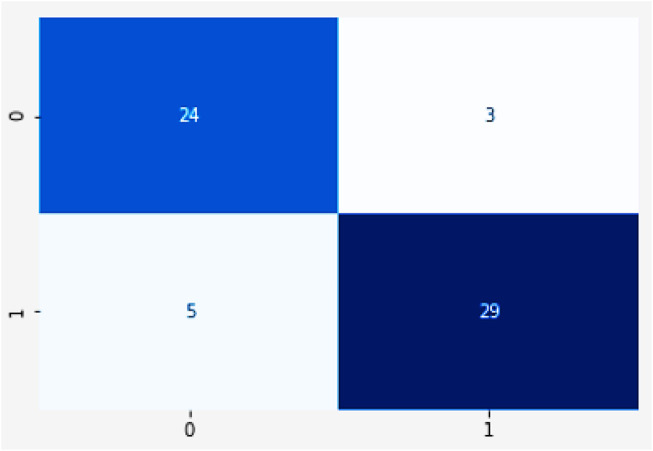
Confusion matrix.

The importance score of a given function or variable is computed by highlighting its significance in relation to the outcome. It also displays the associated functions and how these can influence the outcome. Figure 18a shows the accuracy analysis (attains an accuracy of 99.2) and Figure 18b shows the loss of test set, has lowest loss rate of 0.1. Figure 19 shows the confusion matrix of our proposed framework. The graph shows the number of times the model was able to correctly predict the absence of cardiovascular disease. It also displays the number of false negatives and positive results.

### 4.4 Comparison analysis

**FIGURE 20:**
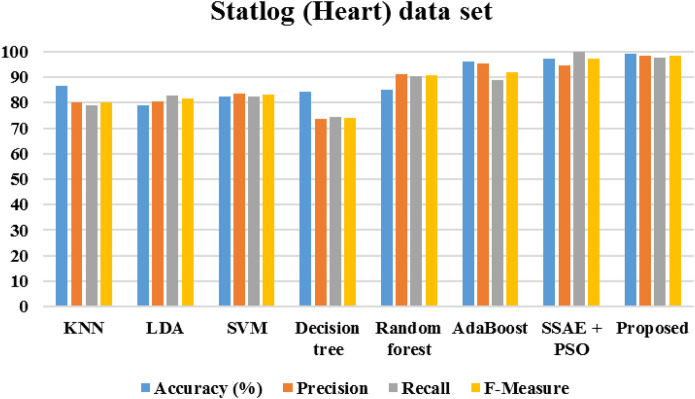
Performance of Statlog (Heart) data set.

**FIGURE 21:**
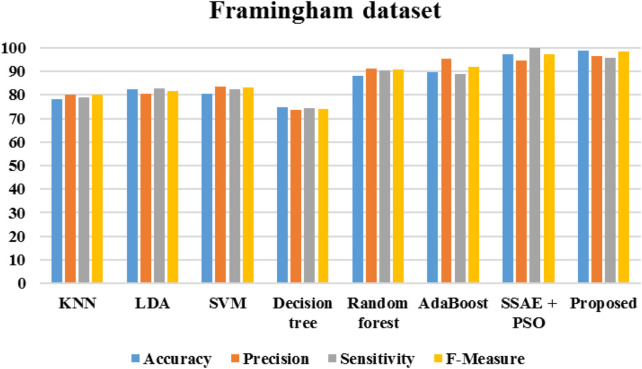
Performance of Framingham data set.

**FIGURE 22:**
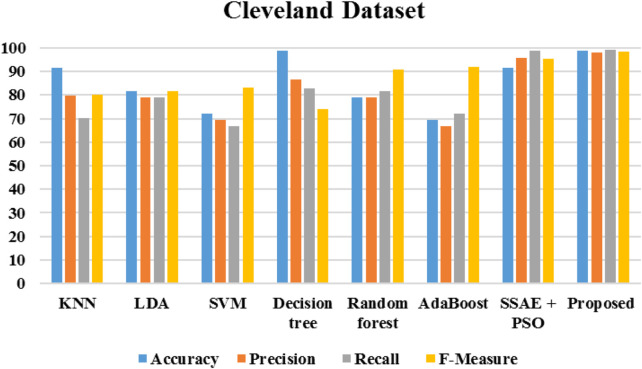
Performance metrics of Cleveland data set.

Figure 20 demonstrates the outcomes of the proposed evaluation of the Statlog (Heart) data set. The following Figure 21 depicted the proposed evaluation of the Framingham data set. Figure 22 shows the proposed outcomes of the Cleveland data set’s evaluation. The proposed evaluation aims to identify relevant risk factors using the proposed detection model and various non-hybrid techniques. The proposed algorithm is mainly focused on extracting features from a specific data set. The results show that a special feature combination is used to perform the extraction. The proposed method is unable to extract crucial data attributes from the information set. This is a significant limitation of the approach. It also has poor portability and accuracy. The traditional method of classification uses various features extraction methods. Deep learning can also perform the same task without requiring the use of any additional features. The paper presents a deep learning model that can independently classify a network. It can also determine its structure. It is then evaluated on three data sets. The proposed algorithm can fully extract the deep-level characteristics of the ECG data to classify heart diseases in complex environments. Even with the same dataset, the precision, recall, and f-measure of the proposed method are better than those used in other applications.

### 4.5 Performance comparison with existing approaches

The algorithms were modeled by using the Framingham dataset and trained using the suggested approach. The recommended method had a sensitivity of 95.7 and an accuracy of 98.7 as presented in Table 1. On the other hand, Table 2 shows the performance of the same algorithms with the Cleveland dataset. The suggested algorithm was able to achieve a sensitivity of 99.2, an accuracy of 98.9, and an F-measure of 98.5. Compared to the other algorithms, it performed better in both cases. But, it is not clear if this method is better suited to handle only two datasets. To test its robustness, we used the exiting frameworks presented in Table 3.

**TABLE 1:** Performance of the various algorithms that take advantage of the Framingham dataset (Framingham heart study, 2025).

Method	Accuracy (%)	Precision (%)	Recall (%)	F-measure (%)
KNN	78.3	80.1	78.9	80.0
LR	83.8	83.9	84.1	83.9
LDA	82.5	80.6	82.8	81.7
SVM	80.5	83.7	82.3	83.0
Decision tree	74.9	73.5	74.5	73.9
Softmax classifier	79.4	80.3	78.6	79.4
XGBoost	91.8	93.8	97.2	95.5
Random forest	88.3	91.1	90.3	90.7
AdaBoost	89.5	95.5	88.9	92.0
SSAE + PSO	97.3	94.8	100.0	97.3
Proposed DBN + SAE + SVM	98.7	98.5	95.7	98.5

**TABLE 2:** Performance of the various algorithms that take advantage of the Cleveland dataset (Cleveland Heart Study dataset available).

Method	Accuracy (%)	Precision (%)	Sensitivity (%)	F-measure (%)
KNN	62.4	60.8	59.4	60.1
LR	78.3	79.0	78.1	78.5
LDA	78.1	80.4	79.2	79.8
SVM	79.6	80.0	78.9	79.4
Decision tree	71.0	69.9	70.8	70.3
Softmax classifier	73.8	71.5	70.0	70.8
XGBoost	87.5	86.4	94.0	90.0
Random forest	86.8	91.4	88.7	90.0
AdaBoost	87.7	93.2	86.5	89.7
SSAE + PSO	96.1	93.0	98.8	95.8
Proposed DBN + SAE + SVM	98.9	98.1	99.2	98.5

**TABLE 3:** Performance of the algorithms against the standards of previous studies related to heart disease.

Author(s)	Method	Accuracy (%)	Precision (%)	Sensitivity (%)	F-measure (%)
Mohan et al. (2019)	Hybrid-Random forest	88.4	90.1	92.8	90.0
Haq et al. (2018)	Improved-Logistic Regression + Feature Selection	89.0	—	77.0	—
Repaka et al (2019b)	Naïve Bayes + Feature Selection	89.77	—	—	—
Samuel et al. (2017)	Fuzzy Analytic Hierarchy Process (AHP) + Artificial Neural Network (ANN)	91.0	—	—	—
Ali et al. (2019)	Optimized Support Vector Machine	92.2	82.9	100.0	—
Li et al. (2020)	Feature Selection + Support Vector Machine	92.3	-	98.0	—
Fitriyani et al. (2020)	XGBoost + Resampling	98.4	98.5	98.3	98.3
Bharti et al. (2021)	Deep learning	94.2	83.1	82.3	—
Zhang et al. (2021)	Linear Support Vector Classification + Deep Neural Network	98.5	99.3	97.8	98.3
Gao et al. (2021)	Bagging ensemble learning method with decision Tree	98.6	—	—	—
Pereira et al. (2016)	Ensemble Convolutional Neural Network + BAT	92.0	87.0	90.0	—
Senthilselvi et al. (2020)	Convolutional Neural Network	82.5	—	—	—
Our approach	DBN + SAE + SVM	96.1	93.0	98.8	95.8

Clinical data analysis plays a vital role in determining the presence of heart disease. But, we must not assume that such prediction is probable in the future. Researchers and scientists have been working on developing new methods to improve this prediction. This method could be utilized by healthcare professionals in developing countries to help them make better decisions. This method could be utilized by clinicians to help detect early signs of diseases and provide the necessary treatment and lifestyle changes.

## 5 Conclusion

Heart disease is one of the main causes of fatalities in developing nations. This chronic illness can affect people from various socioeconomic backgrounds. Getting diagnosed early can help prevent heart disease from causing damage. We utilized deep learning to develop a system that could predict the severity of a person’s heart condition. The system was evaluated on three datasets: Statlog (Heart), Cleveland, and Framingham. It was trained on full features and optimized for its various features. The evaluation metrics were utilized to assess the effectiveness of the system. These included sensitivity, specificity, and F measure. The system performed well on all three datasets, achieving an overall accuracy of 99.2. It also exhibited a sensitivity of 97.5 and a specificity of 98.5.The goal of the next phase of the study is to develop more sophisticated features and classification algorithms that will improve the system’s performance. This involves identifying patterns in the data and using these to improve the predictive capabilities of the system. Further Model Tuning and Cross-Validation are also taken into account. Model tuning involves modifying the algorithm’s parameters to make it perform better. Cross-validation involves evaluating the model in different sets of data to make sure it can generalize well.

## Data Availability

The original contributions presented in the study are included in the article/supplementary material, further inquiries can be directed to the corresponding author.
